# Association of asthma exacerbations with paper mulberry *(Broussenetia papyrifera)* pollen in Islamabad: An observational study

**DOI:** 10.7189/jogh.13.04091

**Published:** 2023-09-01

**Authors:** Osman M Yusuf, Aimal T Rextin, Bakhtawar Ahmed, Rubina Aman, Tanveer Anjum, Saqib Mustafa, Mehwish Nasim, Shahida O Yusuf, Chun Lin, Summan Zahra, Hillary Pinnock, Jürgen Schwarze

**Affiliations:** 1 The Allergy & Asthma Institute, Islamabad, Pakistan; 2 National University of Science and Technology, Islamabad, Pakistan; 3 Pakistan Institute of Medical Sciences, Islamabad, Pakistan; 4 The University of Western Australia, Crawley, Western Australia, Australia; 5 NIHR Global Health Research Unit on Respiratory Health (RESPIRE), Usher Institute, The University of Edinburgh, Edinburgh, United Kingdom; 6 Child Life and Health, Centre for Inflammation Research, The University of Edinburgh, Edinburgh, United Kingdom

## Abstract

**Background:**

Although the role of airborne plant pollen in causing allergic rhinitis has been established, the association of concentrations of paper mulberry *(Broussenetia papyrifera)* pollens in the air and incidence of asthma exacerbations has not, despite an observed increase in the number of asthma patients attending physician clinics and hospital Accident and Emergency (A&E) Departments during the paper mulberry pollen season. We aimed to assess the association between paper mulberry pollen concentrations (typically peaking in March each year) and asthma exacerbations in the city of Islamabad.

**Methods:**

We used three approaches to investigate the correlation of paper mulberry pollen concentration with asthma exacerbations: A retrospective analysis of historical records (2000-2019) of asthma exacerbations of patients from the Allergy and Asthma Institute, Pakistan (n = 284), an analysis of daily nebulisations in patients attending the A&E Department of the Pakistan Institute of Medical Sciences (March 2020 to July 2021), a prospective peak expiratory flow rate (PEFR) diary from participants (n = 40) with or without asthma and with or without paper mulberry sensitisation. We examined associations between pollen data and asthma exacerbations using Pearson correlation.

**Results:**

We found a strong positive correlation between mean paper mulberry pollen counts and clinical records of asthma exacerbations in patients sensitised to paper mulberry (Pearson correlation coefficient (r) = 0.86; *P* < 0.001), but not in non-sensitised patients (r = 0.32; *P* = 0.3). There was a moderate positive correlation between monthly nebulisation counts and pollen counts (r = 0.56; *P* = 0.03), and a strong negative correlation between percent predicted PEFR and pollen counts in sensitised asthma patients (r = -0.72, *P* < 0.001). However, these correlations were of low magnitude in the non-sensitised asthma (r = -0.16; *P* < 0.001) and sensitised non-asthma (r = -0.28; *P* < 0.001) groups.

**Conclusions:**

Our three approaches to analysis all showed an association between high paper mulberry pollen concentration in Islamabad and asthma exacerbations. Predicting pollen peaks could enable alerts and mobilise strategies to proactively manage these peaks of asthma exacerbations.

An estimated 339 million people suffer from asthma worldwide [1,2], including 4.3% of the population of Pakistan [3]. Depending upon individual susceptibility, asthma can be exacerbated by various triggers, like air pollutants, viral respiratory infections, and aeroallergens such as fungal spores and pollen grains [4,5]. Other factors that affect pollen-asthma exacerbations include precipitation [6], humidity [7,8], thunderstorms [9-11], wind [12,13], periods of dry weather [14], and heat [15].

Studies carried out in different countries have found a correlation between allergenic pollen counts, asthma exacerbations, and asthma-related Accident and Emergency (A&E) Department visits in both adults and children [5,9-11,13,16]. “Pollen calendars”, which indicate the variety of local pollen types present in the atmosphere and the duration and severity of the pollen season, facilitate the management of pollen allergies and asthma, allowing individuals with pollen allergy to take timely preventive measures [17].

Two main pollens have been implicated in seasonal allergies in Islamabad: paper mulberry (*Broussonetia papyrifera*) during March/April and cannabis (*Cannabis sativa*) in July to September [18]. Paper mulberry trees release large quantities of pollen in spring, reaching a peak of about 40 000 pollen grains/m^3^ in a single day [19]. Temporal association over many years [20], clinical observations [21,22], and scientific plausibility [23,24], have fuelled discussions that exacerbations of allergic rhino-conjunctivitis and asthma, including increased number of hospital admissions in the months of March and April [25], result from the high concentrations of paper mulberry pollen. Due to the significant annual disruption and fear among individuals with asthma [26], we aimed to investigate if the high concentrations of paper mulberry pollen in Islamabad cause the seasonal asthma exacerbations.

Consequently, we first intended to test potential methodologies for future, fully powered studies seeking to determine a possible causal link between high levels of paper mulberry pollen and asthma exacerbations. Simultaneously, we aimed to identify likely challenges of such a study and determine whether there is sufficient evidence of an association to warrant further investigation.

## METHODS

### Study design

We used three approaches to investigate the correlation of paper mulberry pollen concentration with asthma exacerbations:

− Retrospective analysis of historical records of patients from the Allergy and Asthma Institute, Pakistan (AAIP), Islamabad;
− Analysis of daily nebulisations in patients attending the A&E Department of the Pakistan Institute of Medical Sciences (PIMS), Islamabad;
− Prospective peak flow diary from participants with or without asthma and with or without paper mulberry sensitisation.

### Data sets

#### Pollen data

We used the publicly available daily counts of eight types of airborne particles (including paper mulberry pollen) from the Pakistan Metrological Department for all analyses [27,28].

#### Coronavirus disease 2019 data

We conducted this study during the coronavirus 2019 (COVID-19) pandemic, so we correlated the changes in daily respiratory data (nebulisations and peak flows) with the number of new COVID-19 cases to differentiate COVID-19-related changes in respiratory data from those caused by paper mulberry pollen allergy.

#### Retrospective analysis of clinical records

We extracted anonymised data from paper-based historical records of AAIP from 2000-2019. AAIP maintains detailed paper-based patient records, which include a detailed history and examination taken on a patient’s first visit and clinical notes, including examination on subsequent visits. We initially intended to perform a time series analysis, but we observed that some patients visited AAIP regularly during this time interval and others just visited once, resulting in the number of data points being too sparse. We note here that, although there are methods to handle missing data in a time series analyses, they work by estimating missing values [29,30], making it unfeasible to apply such a method due to the sparsity of data points. We further evaluated if monthly averaging and then applying time series would be meaningful, but we observed that time series analyses on monthly averages would be unfeasible, as monthly averaging will smooth out the variations in important cofounders including weather and holidays. Consequently, we could not apply time series analysis and instead opted for a χ^2^ test [31]. Out of all eligible patients with a history of asthma and who had undergone skin prick testing for standard aeroallergens, we selected a random sample of 284 patients. We calculated the sample size based on a 95% confidence level with 30% estimated prevalence of paper mulberry pollen sensitisation, giving a statistical power of 94.6% for the χ^2^ hypothesis test.

We collected data about patients’ asthma, which contained three types of information about asthma exacerbations:

− Previous history: The months when the patient reported that they suffered from asthma symptoms, as recorded in the clinical history by the physician on the patient’s first visit;
− Patient-reported asthma exacerbations: The months in which the patient reported that they had suffered from troublesome asthma symptoms (including cough, wheeze and/or shortness of breath, relieved by reliever medication) since their previous clinic visit.
− Physician-observed asthma exacerbations: The calendar date when patients attended AAIP for care for uncontrolled asthma confirmed by a physician following examination. A patient was confirmed as having an asthma exacerbation if the physician noted a reduction of 20% or more of their predicted peak expiratory flow rates (PEFR) during the examination and if this was reversible with salbutamol inhalation.

We labelled a patient as sensitised to paper mulberry pollen if the wheal size to the skin prick test was larger than 5 mm.

#### Analysis of daily nebulisations

We collected anonymised daily record of patients attending the A&E Department at PIMS, Islamabad, who were labelled “status asthmaticus” after clinical assessment by an A&E physician and/or a pulmonologist, after which they received nebulisation with bronchodilators. These data were collected from 1 March 2020 until the end of July 2021; we were unable to collect some data due to COVID-19-related restrictions. The longest such interval stretched from 11 April 2020 to 6 July 2020.

#### Prospective peak flow diaries

The third data set were daily peak flow measurements from a cohort of 40 adult patients diagnosed at AAIP and with a record of skin prick tests to common local allergens. Clinical staff at the AAIP provided potential participants with study information and gave them time to consider participation before providing informed written consent.

Participants were provided with peak flow meters and asked to record three readings every morning from 1 March 2020 to 31 July 2021, if applicable, before taking a bronchodilator. The best reading on each day was used for analysis. There were ten patients per each of the following four categories:

1. Sensitised asthma: Participant with asthma who were sensitised to paper mulberry pollen;
2. Non-sensitised asthma: Participant with asthma who were not sensitised to paper mulberry pollen;
3. Sensitised non-asthma: Participant without asthma who were sensitised to paper mulberry pollen;
4. Non-sensitised non-asthma: Participants without asthma who were not sensitised to paper mulberry pollen.

### Statistical analysis

We performed all exploratory and inferential analyses using R, version 4.3.0. (R Core Team, Auckland, New Zealand). 

We analysed the three data sources by correlating asthma related health outcomes with pollen count data using Pearson correlation. As the input data for asthma symptoms were categorical (i.e. yes/no), we used the χ^2^ to assess differences between asthmatic episodes of sensitised and non-sensitised patients. We considered *P* < 0.05 as statistically significant.

We did not calculate sample sizes, as this was a feasibility study. We considered a sample size of 40 participants sufficient to demonstrate feasibility and provide insight into likely outcomes, achievable within the available budget.

## RESULTS

### Pollen data

The pollen data collected by the Pakistan Meteorological Department over 17 years (2003-2019) showed that paper mulberry pollen starts being released at the beginning of March and reaches its peak around the third week or early fourth week of March, with maximum daily pollen counts of 35 000 to 40 000 grains/m^3^, which begin to fall rapidly by the start of April. We therefore considered March as the peak pollen season for the retrospective analysis of data from clinical records.

### Retrospective analysis of clinical records

#### Participants

From 946 eligible patients in the AAIP database, we randomly selected 284, 46.7% of whom were female; 9.9% were <18 years old, 53.3% were 19-35 years old, 35.3% were 36-65 years of age, and 1.5% were >65 years old. In this sample, 118 (41.5%) patients were sensitised to paper mulberry pollen, while the remaining 166 (58.5%) were not. The participants visited AAIP for a clinical review on average 4.40 (standard deviation (SD) = 1.33) times over the 19 years (range = 1-28 visits).

We compared the number of sensitised patients with a record of an asthma exacerbation in March to non-sensitised patients using the χ^2^ test with *P* < 0.05.

#### Incidence of asthma exacerbations in March

Regarding previous history recorded during first attendance, 102 out of 118 (85.0%) of patients sensitised to paper mulberry pollen reported a history of asthma exacerbations in March compared to 105 out of166 (63%) patients not sensitised to paper mulberry (χ^2^ = 18.77; *P* < 0.001). Regarding recent asthma exacerbations in March during follow-up, 34 (28.8%) of the sensitised patients reported an asthma event at least once during March compared to 10 (6.0%) for the non-sensitised patients (χ^2^ = 27.36; *P* < 0.001). Overall, 25 (21.0%) of paper mulberry sensitised patients had a physician-confirmed episode of an asthma exacerbation in March compared to 5 (3.0%) of non-sensitised patients (χ^2^ = 24.1; *P* < 0.001).

#### Correlation of asthma exacerbations with pollen counts

To investigate if asthma exacerbations and pollen counts were correlated over the entire timespan of the data, we planned to correlate the monthly time series of asthma episodes over time from 2000 to 2019 for each of the 284 patients with the pollen count record for the same duration. However, this was not possible because the patients were not examined every year, as they only visited AAIP when they needed medical advice.

**Figure 1:**
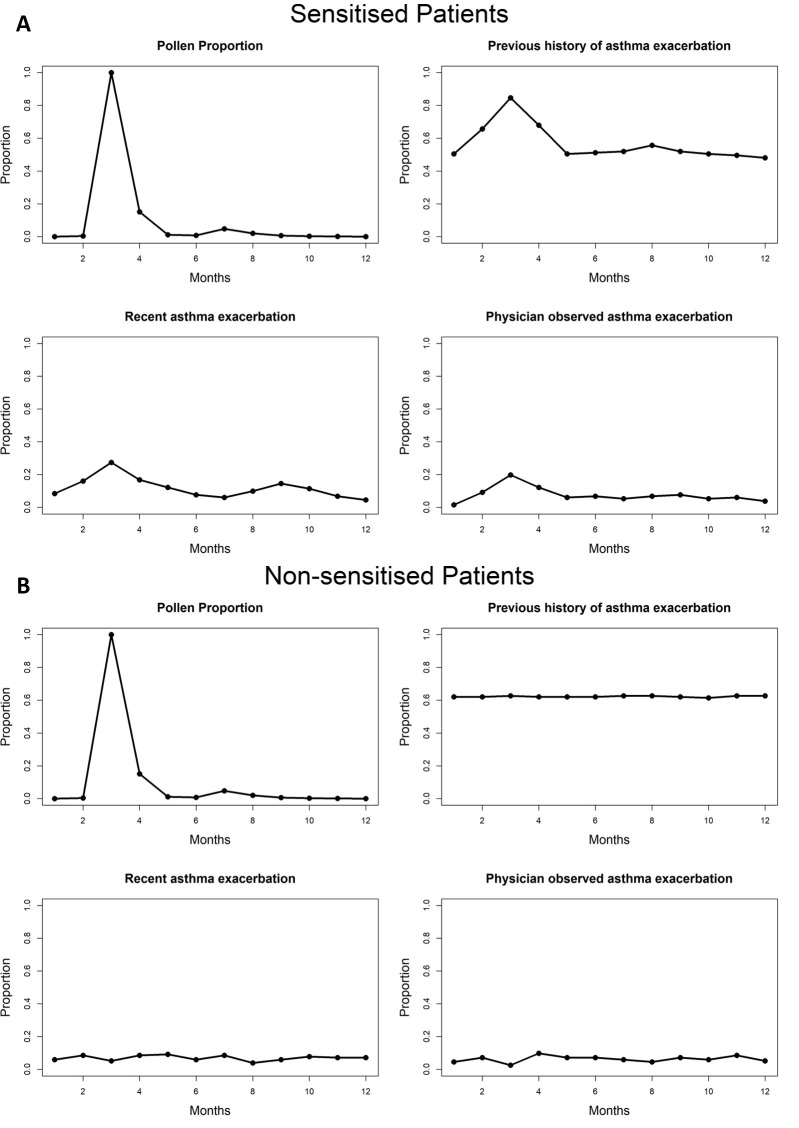
Comparison of mean asthma exacerbations and mean paper mulberry pollen counts for sensitised and non-sensitised patients. **Panel A. **Patients with paper mulberry sensitation. **Panel B.** Patients without paper mulberry sensitization. Both groups reported the months of asthma exacerbations retrospectively at their first clinic visit (previous history) and at subsequent clinic visits (recent asthma exacerbations). Furthermore, the month of a physician-confirmed asthma exacerbation and paper mulberry pollen counts were documented. All data were normalised.

To overcome this lack of data, we correlated the mean values of pollen counts and asthma incidence in each month over all the years. We normalised the pollen counts and plotted the number of asthma episodes between 0 and 1, which showed that asthma episodes for sensitised patients rose with increasing paper mulberry pollen counts, but not for non-sensitised patients (**Figure 1**). The Pearson correlation values showed a strong positive correlation of high magnitude between paper mulberry pollen counts and asthma episodes in patients sensitised to paper mulberry, but not in patients who are not sensitised (**Table 1**).

**Table 1:** Pearson correlation values of average monthly asthma exacerbations and average monthly pollen counts

Patient type	Previous history of reported asthma exacerbations	Recent asthma exacerbations reported at follow-up visit.	Physician-observed asthma exacerbations:
Paper mulberry sensitised	*R* = 0.86; *P* < 0.001	*R* = 0.81; *P* = 0.001	*R* = 0.87; *P* < 0.001
Not paper mulberry sensitised	*R* = 0.32; *P* = 0.3	*R* = -0.29; *P* = 0.35	*R* = -0.52; *P* = 0.08

*R* – Pearson correlation coefficient

### Analysis of bronchodilator nebulisations at A&E

#### Data

We collected daily numbers of bronchodilator nebulisations at the A&E Department at PIMS, Islamabad from 1 March 2020 to 30 July 2021 (517 days). These counts were not possible on 160 out of 517 days (31%) due to COVID-19 restrictions and illness preventing our data collectors from attending A&E. The longest phase without nebulisation counts lasted 87 days (11 April 2020 to 6 July 2020).

#### Correlation of nebulisation with pollen counts

**Figure 2:**
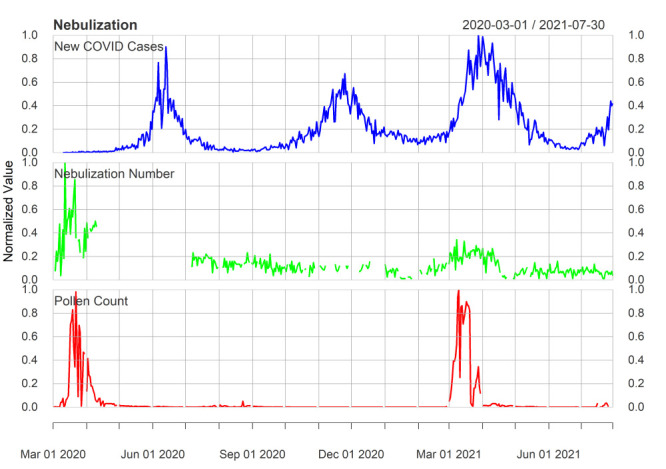
Comparison of daily numbers of nebulisations, daily pollen concentration and daily new confirmed COVID-19 infections. All data were collected from 1 March 2020 to 30 July 2021. The blue lines show the new infections on a day, the green line show the nebulisation number at A&E at PIMS, while the red line shows the pollen count on that day. Missing values are shown by gaps in the line.

The numbers of daily nebulisations, daily paper mulberry pollen counts, and new cases of COVID-19 infection were normalised (between 0 and 1) and then correlated (**Figure 2**). The daily number of nebulisations increased with rising paper mulberry pollen concentrations, but seemed to be independent of the daily new COVID-19 infections. This is exemplified in the peak of new cases of COVID-19 in December 2020, when daily nebulisation numbers remained low.

Statistically, however, due to sudden fluctuations in the numbers and because of missing values in the nebulisation data, we could not directly correlate these data [31]. We thus computed monthly averages and then applied Pearson correlation; we found a positive correlation of moderate magnitude between nebulisation counts in each month and pollen counts (Pearson correlation coefficient (r) = 0.56; *P* = 0.03) but not between number of nebulisations and daily COVID-19 cases (r = -0.22; *P* = 0.43).

### Analysis of peak flow readings in the cohort

#### Participants

Out of the 40 initial participants, five patients, one from the non-sensitised asthma and four from the sensitized non-asthma group, did not collect any data and were excluded from analyses. The remaining 35 participants provided data on 518 days (**Table 2**).

**Table 2:** Patient characteristics and correlations of paper mulberry pollen counts with mean percentage of expected PEFR values

	Sensitised asthma (n = 10)	Non-sensitised asthma (n = 9)	Sensitised non-asthma (n = 6)	Non-sensitised non-asthma (n = 10)
Age in years, mean (SD)	42.3 (12.0)	40 (10.3)	35.2 (4.8)	30.4 (9.2)
Sex	60% male, 40% female	44.44% male, 55.56% female	50% male, 50% female	60% male, 40% female
Percent of submitted readings	97.1%	90.93%	90.73%	82.24%
Correlation of PEFR with pollen counts	r = -0.72; *P* < 0.001	r = -0.16; *P* < 0.001	r = -0.28; *P* < 0.001	r = -0.03; *P* = 0.56

SD – standard deviation, r – Pearson correlation coefficient, PEFR – predicted peak expiratory flow rate

#### Correlation of daily peak flow values with pollen counts

**Figure 3:**
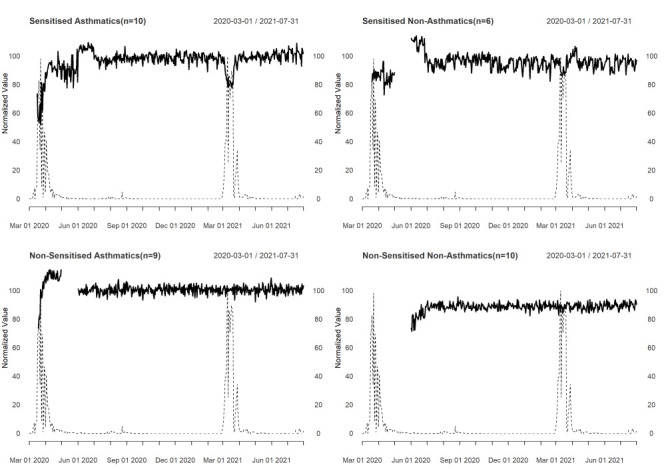
Comparison of mean percentage of expected PEFR with daily paper mulberry pollen counts. The solid thick black line shows the mean percentage of expected PEFR, while the thin dashed line shows the normalized daily paper mulberry count. Breaks in the PEFR data show times when none of the participants of a group provided data.

We plotted the daily mean percentage of predicted PEFR against the daily pollen counts (**Figure 3**). In both groups of asthma patients and in sensitised patients without asthma, the mean expected percentage of PEFR dropped with rising pollen concentrations. This was not the case for non-sensitised non-asthmatics. We found a negative correlation of high magnitude between predicted PEFR and pollen counts in sensitised asthmatics, while these correlations were only of low magnitude in non-sensitised asthmatics and sensitised non-asthmatics (**Table 2**).

**Figure 4:**
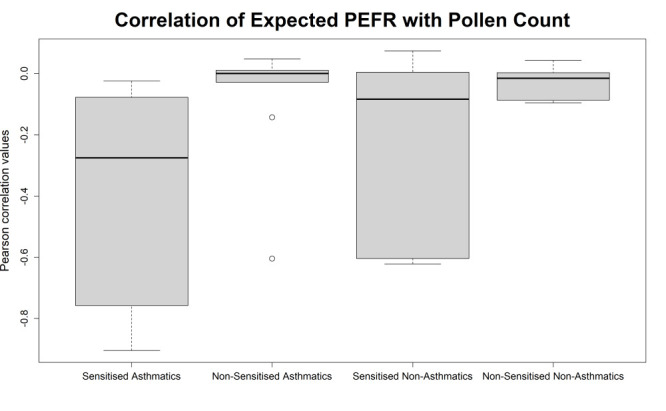
Pearson’s correlation coefficients between percent expected PEFR of individual asthma patients and paper mulberry pollen counts. Individual correlations between percent expected PEFR and paper mulberry pollen counts were calculated and are presented as box plots (box = 25^th^ to 75^th^ centile, thick black line = median, lower whiskers = up to 24^th^ centile, upper whiskers = above 75^th^ centile, dots = outliers).

As each patient may respond differently to high paper mulberry pollen, we calculated the Pearson correlation between daily percentage of expected PEFR reading and pollen concentration on that day for each participant. To visualise the holistic effect of high pollen concentrations, we plotted the resulting individual correlation values as a boxplot (**Figure 4**) to show the spread in the strength of PEFR/pollen count correlation amongst patients. The figure indicates a close link between high level paper mulberry pollen exposure and compromised pulmonary function in both groups of sensitised patients, but not in the two groups of non-sensitised patients. We noted that the data of the two groups were not approximately normally distributed, so we used the Mann-Whitney U test with a *P* < 0.05, applying the test after merging the two groups of sensitised individuals into one and two groups of non-sensitised individuals into another group. Our null hypothesis was that there is no significant difference between the medians of the sensitised groups and the non-sensitised groups, and our alternative hypothesis was that here is a significant difference between the medians of the two independent groups. We obtained a *P*-value of 0.003, suggesting a statistically significant difference between the two groups.

## DISCUSSION

Islamabad reportedly has one of the highest airborne pollen counts of paper mulberry pollens globally, with values exceeding 40 000 pollen grains/m^3^ in March. Airborne pollen concentration is affected by various environmental factors such as temperature, rainfall, cloud cover, the intensity of sunlight, and wind speed [32]. This observational feasibility study found associations between concentrations of paper mulberry pollen in Islamabad and asthma exacerbations, using a combination of retrospective and prospective data. Patients sensitised to paper mulberry pollen, as diagnosed by skin prick testing, were more likely to report a previous history of asthma exacerbations in March, the month with the highest paper mulberry pollen counts. Similarly, a greater number of paper mulberry pollen patients reported worsening asthma symptoms in March on follow-up visits compared to non-sensitised patients. We found a similar positive correlation between physician-observed asthma exacerbation, daily nebulisation counts, and pollen concentration. The monitored PEFR decreased in participants with asthma and paper mulberry pollen sensitisation and, to some degree, in sensitised patients without asthma, with the magnitude of negative correlation being highest in the sensitised-asthma group. These findings suggest a correlation and agree with our initial hypothesis that the rise in pollen concentration of paper mulberry is linked to asthma exacerbations in paper-mulberry pollen sensitised residents of Islamabad, enabling us to better understand the magnitude and quality of this relationship.

### Discussion in relation to previous publications

Our results are consistent with previous findings which link ambient concentration of allergenic pollen to exacerbation in asthma symptoms. A systematic review by Shrestha et al. [33] showed that an increase in pollen concentrations increased asthma exacerbations and asthma-related hospitalisations in children and adolescents. Using the Melbourne Air Pollen Children and Adolescent Health (MAPCAH) study, Batra et al. [34] found an increased risk of asthma hospital admissions two days after exposure to pollen and fungal spores during their peak periods. Similarly, Idrose et al. [35] found that pollen-sensitised adults with asthma experienced a decrease in lung function two to three days after exposure to high levels of grass pollen. In New York, increases in asthma-related A&E department visits and asthma-related hospitalisations were found to be closely related to peaks in pollen counts in the spring season [36,37]. In Brussels, Belgium, grass, birch, and hornbeam pollen caused asthma-related adverse effects in individuals of less than 60 years of age [38]. A study carried out in London demonstrated the concept of “lag time” between exposure on “very high” pollen days and increased asthma hospital admissions two to five days later [39]. Yoshida et al. [40] showed that sensitised Japanese school children develop more asthma symptoms during the cedar pollen season than in the non-pollen season. In Kolkata, a densely populated city, pollen counts and asthma-related A&E visits peaked during July and September [41]. In New South Wales, Australia, presentations in A&E departments increased during the rye grass pollination season (October- November) [42]. Thus, the rye grass pollen peak in spring can results in debilitating asthma exacerbations leading patients into A&E units. Significant correlations between increasing rye grass pollen counts and hospital visits have also been demonstrated in other studies in both adults and children [43,44].

Our results can help inform effective strategies for the prevention and management of asthma exacerbation during periods of increased pollen concentrations. Pollen allergy considerably impairs quality of life, sleep, school performance, and productivity [5,6,10]. In a developing country like Pakistan, the employment and sick leave conditions are not as favourable as in high income countries, and many people cannot afford to miss work, especially those working on a casual or daily wage basis. It is essential to publicly disseminate information regarding patterns of paper mulberry pollen release and to raise awareness of appropriate preventive measures, such as regular use of asthma preventer medication (e.g. inhaled corticosteroids) during the peak season, limiting time outdoors on high pollen days, performing saline irrigation of nasal passages [45], and always having access to asthma rescue medication. Interestingly, we observed that paper mulberry pollen-sensitised asthma patients who took their medication regularly during the peak pollen season maintained stable PEFR values while these dropped substantially in four out of five patients who did not use their medication (data not shown).

Pollen calendars will be essential in informing patients, healthcare professionals, and other relevant stakeholders on when to start using preventive measures. Masks, which are already widely used to reduce viral spread in the COVID-19 pandemic, could play an important role in reducing paper mulberry pollen-induced asthma exacerbations. There is a lack of studies quantifying the impact of timely preventive interventions in reducing the burden on health care and improving quality of life by reducing asthma exacerbations during the pollen season. Further studies and public engagement are needed to provide better preventive strategies prior to the pollen season to prevent asthma exacerbations.

### Strengths and limitations

This is the first detailed analysis of patients with and without asthma who are, or are not, paper mulberry pollen sensitised. Our study adds to previous work by using multiple data sets, i.e. retrospective clinical patient data, nebulisation data from A&E, and prospective PEFR readings directly from patients, allowing multidimensional assessment of the association between asthma exacerbations and pollen counts. Previous studies, in contrast, have only established a correlation between asthma exacerbations and paper mulberry pollen concentrations, assessed through the number of hospital visits [10,11].

However, our results must be interpreted with caution due to some data limitations; the severe impact of the COVID-19 pandemic, which will have affected patient behaviour, the background attack rate, and our ability to collect data. The COVID-19 pandemic was still in its initial stages in March 2020 and the number of COVID-19 cases was low until the first infection wave in Islamabad in May 2021, which was after the paper mulberry pollen season. Nebulisation could not be recorded for the months of May and June 2020 due to COVID-19 emergencies in hospitals, resulting in cessation of research activities. As people became more aware and more cases of COVID-19 were recorded in 2021, the increased use of masks and lockdowns may have been responsible for fewer nebulisations in March 2021. Lack of awareness and the general fear of the disease may also have contributed to more people seeking emergency hospital care in March 2020. Increases in COVID-19 cases in winter 2020 and May 2021 were not associated with increased hospital visits for nebulisations, resulting in an absence of correlation between these parameters.

Furthermore, our retrospective data collection may have been affected by recall bias, but the results are consistent with our findings of increased A&E visits for nebulisation during the months of March 2020 and March 2021. We also could not assess the effect of weather variables such as rain, temperature, and wind speed. A key issue, common to much environmental epidemiology research, is that of direct environmental exposure assessment for pollen and potential confounders. Paper mulberry pollen counts were measured at two different sites in Islamabad, and the measurements were assumed to apply to entire urban Islamabad. In coming years, climate change may lead to significant changes in the quantity and pattern of production and release of pollen grains by different flowering species, including paper mulberry pollen.

Regarding the PEFR cohort, we recognise the limitations of the small sample size; however, this was a feasibility study, and the sample size was pragmatic to allow assessment of feasibility, to be manageable within a limited budget, and was not based on a power calculation.

## CONCLUSIONS

While the link between high pollen counts and seasonal asthma exacerbations, assessed as hospital visits, has previously been established, there was a lack of empirical evidence establishing a definitive association between an increase in paper mulberry pollen concentration and deterioration of pulmonary function. Using data from retrospective records of patients, from A&E physicians, and PEFR measurements, we found a significant increase in reported asthma exacerbations and A&E department visits with asthma-related nebulisations and a decrease in pulmonary function with rising concentrations of paper mulberry pollens. These results are in line with previous studies on this subject and offer evidence of the burden placed on patients and health care by seasonal increases in pollen counts. Besides developing pollen calendars to predict the rise in pollen concentrations in advance, our study also offers a basis for new research to quantify the impact of paper mulberry pollen-related asthma exacerbation on health care costs and quality of life and to test preventive strategies and their implementation.
